# Anti-lipolysis-stimulated lipoprotein receptor antibody-drug conjugate to treat triple-negative breast cancer

**DOI:** 10.3389/fonc.2025.1663743

**Published:** 2025-09-17

**Authors:** Zhuoxin “Zora” Zhou, Davis Ballard, Tanvi Varadkar, Jiashuai Zhang, Zhantao Du, Ashley George, Aaron Krabacher, Rachel Yengo, Lufang Zhou, Xiaoguang “Margaret” Liu

**Affiliations:** ^1^ Department of Chemical and Biomolecular Engineering, The Ohio State University, Columbus, OH, United States; ^2^ Department of Biomedical Engineering, The Ohio State University, Columbus, OH, United States; ^3^ Comprehensive Cancer Center, The Ohio State University, Columbus, OH, United States

**Keywords:** lipolysis-stimulated lipoprotein receptor (LSR), antibody-drug conjugate (ADC), triple-negative breast cancer (TNBC), targeted therapy, evaluations

## Abstract

Triple-negative breast cancer (TNBC), the most aggressive breast cancer subtype (ER^-^/PR^-^/HER2^-^), is characterized by rapid proliferation, high metastatic rate and frequent recurrence. The development of targeted therapies for TNBC, such as antibody-drug conjugates (ADCs), has been limited by the lack of promising cell surface receptors. Our recent findings revealed that lipolysis-stimulated lipoprotein receptor (LSR) is overexpressed in breast cancer patients. The objective of this study was to develop an anti-LSR monoclonal antibody (mAb) and ADC for TNBC treatment. We observed high transcript and surface expression of LSR across various breast cancer subtypes, with over 63% of TNBC patient tissue samples exhibiting elevated expression. A new mAb targeting the extracellular domain of LSR was developed, engineered, and evaluated *in vitro* and *in vivo*. The ADC, constructed by conjugating LSR mAb with a cytotoxic agent mertansine (DM1), demonstrated potent anti-TNBC cytotoxicity in three cell lines. *In vivo* anti-cancer efficacy was evaluated in two TNBC xenografted mouse models, where a 24 mg/kg-body weight dose of LSR mAb-DM1 reduced tumor burden by 85% in one model and prevented tumor regrowth in the second model. Notably, no off-target effects or systemic toxicity were observed in animal models during or after treatment. This study highlights LSR as a promising therapeutic target and the anti-LSR mAb and ADC as potential targeted therapies for TNBC.

## Introduction

Triple-negative breast cancer (TNBC) is highly aggressive and metastatic (1), with the poorest prognosis among all breast cancer subtypes (2, 3). Standard cytotoxic chemotherapies, such as doxorubicin and paclitaxel, offer limited clinical benefits due to severe side effects, emergence of drug resistance, and high recurrence rates (4–6). As previously reviewed (7, 8), clinical trials demonstrate that immune checkpoint inhibitors, such as the anti-PD-1 pembrolizumab and the anti-PD-L1 durvalumab improve progression-free survival and overall survival in patients with TNBC when combined with chemotherapy, whereas monotherapy shows limited therapeutic efficacy. Sacituzumab govitecan-hziy (Trodelvy), a trophoblast cell-surface antigen 2 (Trop-2)-targeted antibody-SN-38 conjugate, has been approved for relapsed or refractory TNBC (9–12) and other cancers (13–16) following standard therapies. However, the limited efficacy of existing chemotherapy and immunotherapy options underscores an urgent need for novel targeted therapies for TNBC.

Targeted therapies, such as monoclonal antibodies (mAbs) and antibody-drug conjugates (ADCs), have been developed and applied in clinical settings to treat various cancers while minimizing adverse effects on the healthy tissues in patients (17–19). ADCs can carry highly potent drugs and specifically target cancer cells *in vivo* to deliver chemotherapy or other therapies. The mAb component of ADCs can also trigger antibody-dependent cell-mediated cytotoxicity, further enhancing their anti-cancer effect. Compared to traditional mAbs and chemotherapies, ADCs offer improved anti-cancer efficacy and reduced adverse effects. For instance, ado-trastuzumab emtansine (T-DM1, Kadcyla) (20, 21) and fam-trastuzumab deruxtecan-nxki (T-Dax, Enhertu) (22, 23) have been approved for human epidermal growth factor receptor 2 (HER2)^+^ breast cancers. ADCs targeting folate receptor alpha, such as rinatabart sesutecan (NCT05579366), AMT-151 (NCT05498597), and LY4170156 (NCT06400472), as well as those targeting protein tyrosine kinase 7, including LY4175408 (NCT07046923) and DAY301 (NCT06752681), are currently being evaluated in Phase 1 or 1/2 clinical trials for advanced or metastatic TNBC and other cancers (https://clinicaltrials.gov/). These developments indicate that ADCs represent a promising targeted therapeutic strategy for TNBC patients, provided suitable surface receptors are identified and validated.

The lipolysis-stimulated lipoprotein receptor (LSR) is a promising therapeutic target for TNBC. LSR is expressed in the plasma membrane, cytoplasm and nucleus of cancer cells with metastatic signatures (24, 25) and is associated with poor disease-free survival in breast cancer patients (25). LSR supports lipid metabolism, promotes tumor growth, defines cell corners to form tricellular tight junctions in epithelial cells, and contributes to cancer cell proliferation and metastasis (24, 26). Recent studies have reported that LSR downregulation inhibits cell proliferation and invasion in endometrial cancer (27, 28), while its overexpression in gastric and breast cancers correlates with increased lipid uptake, cell proliferation, and cancer stem cell-like features (29, 30). One study using TNBC xenografted mouse models revealed that LSR overexpression has been linked to tumor development (25). One recent study showed that anti-LSR antibody-monomethyl auristatin E conjugate significantly inhibited ovarian cancer growth at high dose (31). Our bioinformatics analysis of The Cancer Genome Atlas (TCGA) dataset and histological analysis of over 100 patient tissues confirmed LSR overexpression in most TNBCs and ER^+^/PR^+^/HER2^+^ breast cancers. Additionally, the anti-LSR mAb developed in this study effectively targeted and delivered chemotherapy to TNBC cells both *in vitro* and *in vivo*.

This study aimed to develop and evaluate an LSR-targeted ADC to treat TNBC. High expression of LSR was confirmed in TNBC cell lines and patient tissues. Leveraging this, a new anti-LSR mAb was developed, engineered and used to conjugate with mertansine (DM1). The mAb’s surface binding, internalization, and tumor-targeting capabilities were validated using flow cytometry, confocal microscopy and *In Vivo* Imaging System (IVIS). The anti-LSR ADC was evaluated *in vitro* through cytotoxicity assays using three TNBC cell lines. Furthermore, its anti-TNBC efficacy was evaluated in two mouse models to assess tumor burden reduction and tumor blockage. Collectively, this study suggests that LSR is a promising therapeutic target for TNBC, and an LSR-targeted ADC may offer a new treatment strategy.

## Materials and methods

Animal studies were conducted according to the Institutional Animal Care and Use Committee (IACUC) Protocol 2022A00000071 that was approved by the university Institutional Biosafety Committee.

### Cell lines and culture media

The human TNBC cell lines MDA-MB-231, MDA-MB-468, and MDA-MB-231-FLuc (ATCC, Manassas, VA, USA) were cultivated in Dulbecco’s Modified Eagle Medium (DMEM) supplemented with 10% fetal bovine serum (FBS, v/v) and 1% penicillin/streptomycin (Pen/Strep, v/v). The mouse TNBC cell lines 4T1 and 4T1-FLuc (ATCC) were maintained in RPMI-1640 medium with 10% FBS and 1% Pen/Strep. The seed train of Expi293F cells for LSR mAb production was maintained in Expi293 Expression Medium containing 4 mM GlutaMAX and 4 g/L glucose. All cell cultures were incubated at 37°C in a humidified incubator with 5% CO_2_ (Caron, Marietta, OH, USA). Media and supplements were obtained from Fisher Scientific (Waltham, MA, USA) or Millipore Sigma (Burlington, MA, USA) unless otherwise specified.

### Development, engineering, and production of anti-LSR mAb

First, the anti-human LSR mAb, targeting the Ig-like V-type extracellular domain (Met1-Asp259) of the surface receptor, was developed using the well-established hybridoma technology as previously described (32–34). Once mAb production was detected in the mice injected with immunogen, the splenocytes were collected and fused with myeloma cells Sp2/0-Ag14 (ATCC) to generate hybridoma cells. Two rounds of single cell cloning were performed to identify the top hybridoma clone with high affinity and mAb titer, quantified via enzyme-linked immunosorbent assay (ELISA) using the extracellular peptide as the coating antigen. Second, the top LSR mAb clone was engineered by fusing its variable region with the Fc region of a human IgG1 for future clinical application.

The engineered LSR mAb was produced from Expi293F cells using transient expression system (Gibco) fed with glucose (6 g/L) and L-GlutaMAX (6 mM). The antibody productions were performed in 2-L stirred-tank bioreactor with process parameters of Temp 37°C, agitation 140 rpm, DO 40% and pH 7.2, or in a 3-L shaker flask at Temp 37°C, agitation 130 rpm, and 8% CO_2_ (33–36). Purification of LSR mAb was performed utilizing a Next Generation Liquid Chromatography system (Bio-Rad, Hercules, CA, USA) following our previous procedures (32–35, 37–40). Specifically, the spent medium containing the mAb product was loaded onto 1-mL or 5-mL Bio-Scale Mini UNOsphere SUPrA affinity chromatography cartridges (Bio-Rad) with buffer A comprised of 0.02 M Na_3_PO_4_ and 0.02 M Na_3_C_6_H_5_O_7_ (pH 7.5). The mAb was eluted with buffer B comprised of 0.1 M NaCl and 0.02 M Na_3_C_6_H_5_O_7_ (pH 3.0).

### Construction of LSR mAb-DM1

The LSR mAb-DM1 ADC was constructed using our previously established conjugation platform (32, 34, 40) with minor modifications. Briefly, the purified LSR mAb was neutralized, desalted via buffer exchange, and concentrated to 5 mg/mL with a 10 kDa MWCO PES concentrator. During the conjugation reaction, 5 mg/mL mAb, 10 mg/mL sulfo-SMCC linker, and 10 mM of DM1 were mixed at a molar ratio of 1:14:18.2. After incubation at 37 °C for 2 hours, the constructed ADC was purified using a Protein A column, desalted using a 10 kDa Slide-A-Lyzer dialysis cassette, and further concentrated with a 10 kDa PES concentrator.

### Characterization of ADCs

The purity and drug-antibody ratio (DAR) of the ADC were analyzed using a High-Performance Liquid Chromatography (HPLC) system (Shimadzu, Columbia, MD, USA) equipped with a MAbPac hydrophobic interaction chromatography (HIC)-butyl column (5 µm, 4.6x100 mm). Two aqueous mobile phases with flow rates of 1.0 mL/min were utilized: Buffer A, 1.5 M (NH_4_)_2_SO_4_ and 50 mM Na_3_PO_4_ at pH 7.0 and Buffer B, 50 mM Na_3_PO_4_ at pH 7.0. UV/Vis spectroscopy was used to confirm ADC conjugation and mass spectrometry was used to quantitate DAR at University Mass Spectrometry and Proteomics facility.

### *In vitro* cytotoxicity

The human and mouse TNBC cells were seeded in 96-well plates at densities of 10,000 cells (MDA-MB-468), 1,000 cells (MDA-MB-231), and 500 cells (4T1) per well. The cells in each well were treated with LSR mAb-DM1 or free DM1 at final concentrations of 0, 2, 4, 10, 20, 50, 100, or 200 nM. After a 5-day incubation at 37°C and 5% CO_2_, relative viability was tested using the MTT Cell Proliferation Assay Kit. The absorbance at 570 nm was measured using a microplate reader (BioTek, Winooski, VT, USA).

### Flow cytometry analysis

The surface binding of engineered LSR mAb to TNBC MDA-MB-231, MDA-MB-468 and 4T1 cells was tested using flow cytometry as described before (41–46). Specifically, the anti-LSR mAb was labeled with fluorescent Alexa Fluor™ 647 kit. Approximately 1×10^6^ (6) TNBC cells were stained with 5 µg of LSR mAb-AF647 or saline (control) at 37 °C for 60 minutes. After washing with PBS three times, the stained cells were analyzed on a BD LSRII flow cytometer (BD Biosciences, San Jose, CA, USA) and data were processed using FlowJo software. The gating was set so that the negative control (i.e. TNBC cells stained with saline) had <0.5% fluorescent population.

### Live-cell confocal microscopy

The internalization of LSR mAb into TNBC cells was assessed using live-cell confocal microscopy. Approximately 10,000 MDA-MB-468 cells were seeded in 35-mm glass-bottom dishes (Cellvis, Mountain View, CA, USA) in 1.5-mL of medium. As described before (47), the cytoplasm of cells was stained with BacMam GFP Transduction Control, and the nucleus was visualized with NucBlue™ Live ReadyProbes Reagent following the manufacturer’s instructions. The LSR mAb labeled with cyanine-5.5 fluorescent dye (Lumiprobe, Hunt Valley, MD, USA) was added to TNBC cultures at a final concentration of 5 µg/mL and incubated for 12 hours. Live-cell images were acquired using an A1R-HD25 confocal microscope (Nikon, Melville, NY, USA).

### *In vivo* imaging system

The 6-week-old NOD SCID gamma (NSG) female mice (Jackson Lab, Bar Harbor, ME, USA) were subcutaneously (s.c.) injected with 10×10^6^ MDA-MB-231-FLuc cells to establish TNBC xenograft models. When tumor volumes reached ~100 mm³, 80 µg of LSR mAb-Cy5.5 was administered intravenously (i.v.) via tail vein. After 24 hours, live-animal imaging was collected at an excitation/emission wavelength of 660/710 nm following intraperitoneal (i.p.) injection of the FLuc substrate luciferin. Mice were then sacrificed to collect major organs, such as brain, heart, lung, spleen, liver and kidneys, for *ex vivo* imaging.

### MDA-MB-231 xenografted models and treatment

A total of 10x10^6^ MDA-MB-231-Flux cells were s.c. injected into 6-week-old female NSG mice (Jackson Lab). Once tumor volumes reached ~50 mm^3^, the mice were randomized into three groups (n = 6) and received i.v. administrations of saline (control), 12 mg/kg ADC, or 24 mg/kg ADC every three or four days (i.e. twice each week). Tumor dimensions were measured with a caliper and volumes were calculated using the formula (length x width ^2^)/2. IVIS imaging was also performed to monitor tumor growth. Mouse body weight was measured twice per week. At the end of the study, all tumor tissues and major organs, such as brain, heart, lung, kidneys, liver, and spleen, were collected for further post-treatment analysis.

### 4T1 xenografted models and treatment

The 6-week-old BALB/cJ female mice were s.c. implanted with 2x10^6^ 4T1-Flux cells per mouse. Tumor growth and body weight were monitored twice each week. When the average tumor volume reached ~50 mm^3^, mice were randomized into two groups (n = 4) and i.v. injected with either saline (control) or 24 mg/kg ADC every three or four days (i.e. twice each week). The study concluded when ulcerations reached 2 mm, after which tumors and organs were collected for further analysis.

### Immunohistochemistry staining

A TNBC patient tissue microarray (TMA, Cat# BR1102, TissueArray, Derwood, MD, USA) and normal organ tissue microarray (Cat# FDA662d, TissueArray) were stained with our engineered anti-LSR mAb following a published IHC staining protocol (47). The stained TMA slides were scanned with a Lionheart FX automated microscope (BioTek, Winooski, VT, USA). LSR expression was quantified with ImageJ and the score was calculated using the formula ((red/blue)_TNBC_/(red/blue)_positive control_)-1)x100. High, medium, and low or no expression levels were defined as scores of >0.5, 0.5 to -0.5 and <-0.5 respectively, using positive control as baseline. Proliferation and apoptosis in the tumor microenvironment after treatment were evaluated using a similar IHC procedure to stain the tissue slides.

### TCGA transcript analysis

The LSR RNA data for breast cancers (TNBC, ER^+^/PR^+^, HER2^+^) and normal breast tissue were obtained from The Cancer Genome Atlas (TCGA) via the UCSC Xena platform (https://xenabrowser.net/datapages/). The raw expression data, originally in fragments per kilobase of transcript per million mapped reads (FPKM), were converted to transcripts per million (TPM) for normalization. Student’s t-test was conducted to assess the differential expression of LSR between various breast cancer subtypes and normal tissue.

### Western blotting

Lysates of TNBC cells were prepared using RIPA buffer and quantified using the Pierce protein assay kit. After separation on a NuPAGE 4-12% gradient SDS-PAGE gel, host cell proteins were transferred onto a PVDF membrane using a power supply (Bio-Rad Laboratories, CA, USA). The PVDF membrane was blocked in 5% non-fat milk in TBST buffer for 1 hour, stained with primary antibodies, i.e. our engineered LSR mAb (2 µg/mL) or anti-β-tubulin antibody (1:1000, Abcam), overnight at 4 °C, and washed three times with TBST. Following the washes, PVDF membrane was incubated with anti-human IgG-HRP (1:5000) or anti-rabbit IgG-HRP (1:3000) in 5% non-fat milk for 1 hour at room temperature. After color development, the washed PVDF membrane was imaged with Azure 300 imaging system (Azure Biosystems, Dublin, CA, USA).

### Hematoxylin and eosin staining

Paraffin-embedded tumor and organ slides were dewaxed in xylene, rehydrated using a graded ethanol series (100% → 50%) and distilled water, and stained with hematoxylin. After dipping in 1% HCl and 70% ethanol, slides were immersed in 1% NH_4_OH for blue color development, followed by a 30-second eosin stain. Finally, the slides were dehydrated in 95% and 100% ethanol and cleared with xylene. Images of the H&E-stained slides were captured using a Lionheart FX Automated Microscope (BioTek, Winooski, VT, USA).

### Statistical analysis

Experimental data were analyzed and presented as mean ± standard error of the mean (SEM). Within-animal changes were evaluated using paired t-tests. Tumor growth and body weight were compared among groups using a two-way ANOVA followed by *post-hoc* (Dunnett’s) analysis. GraphPad Prism was used for statistical analysis, a significance threshold of *P*-value< 0.05 was used for all tests. All adjusted *P*-values were calculated using the Benjamini-Hochberg method to control the FDR.

### Data and materials availability

All data generated during this study are included in the main text of this article. Most materials used in the study, except the developed and engineered LSR mAb, are commercially available. The commercial NSG (NOD.Cg-Prkdc^scid^ Il2rg^tm1Wjl^/SzJ) mice and BALB/cJ mice were purchased from Jackson Laboratory.

## Results

### LSR expression in TNBCs

**Figure 1:**
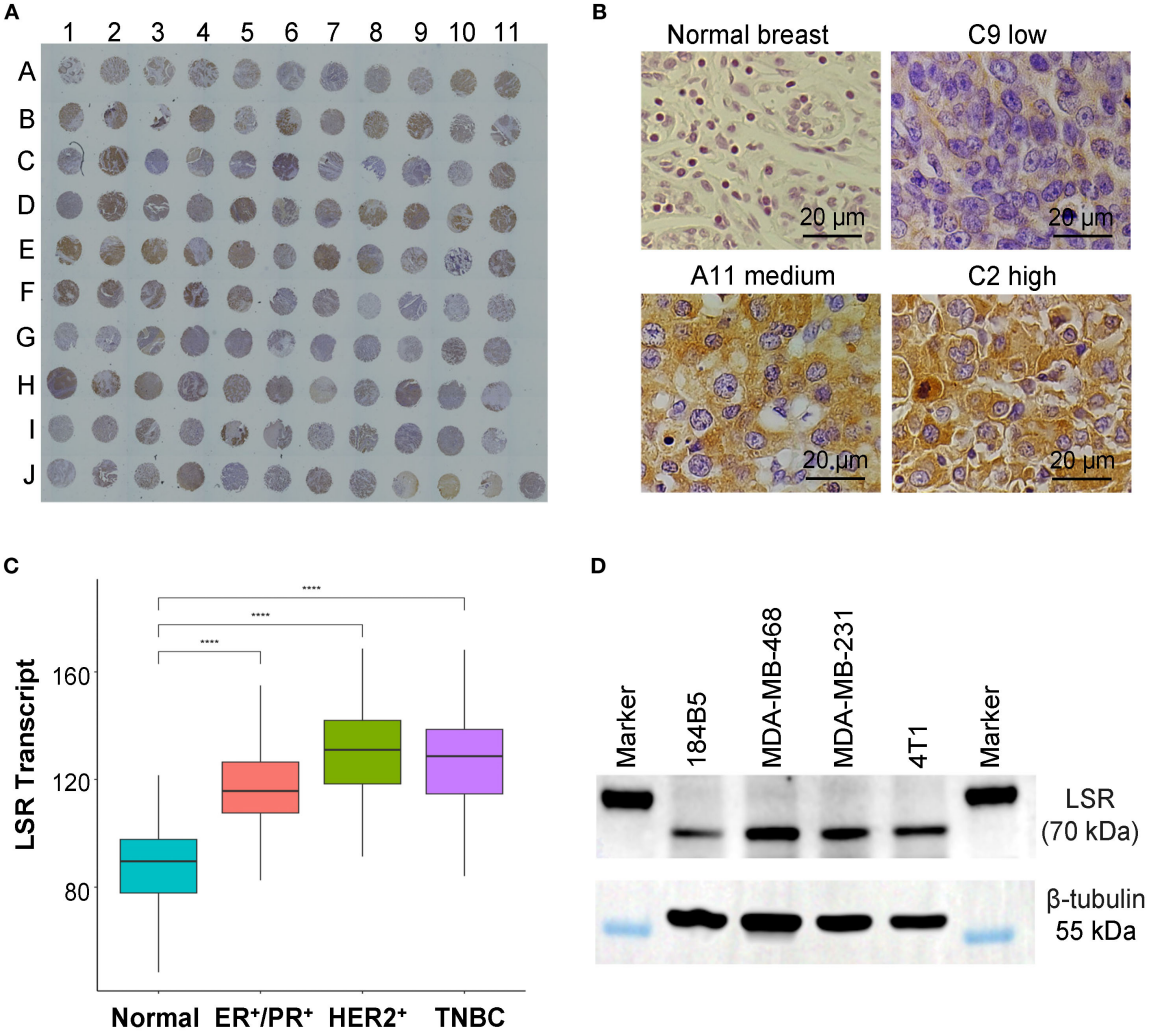
Evaluations of LSR surface receptor in TNBCs. **(A)** IHC staining of TNBC patient tissue microarray (TMA) with our engineered anti-LSR antibody. n = 110. **(B)** Representative images of normal breast and TNBC tissues with low, medium and high levels of LSR expression. **(C)** TCGA transcript analysis of LSR expression of normal breast and breast cancers. **(D)** Western blotting of TNBC cell lines (MDA-MB-231, MDA-MB-468, 4T1) and normal breast epithelial cell (184B5).

To assess LSR protein expression in TNBCs, we performed IHC staining on a patient-derived tumor microarray (TMA, n = 110) using our developed LSR mAb (**Figure 1A**). Representative IHC images of tissue cores displaying varying LSR expression levels are shown in **Figure 1B**. High LSR expression was observed in 35 patient samples (32.7%), and moderate expression in 32 samples (29.9%), collectively accounting for 62.6% of TNBC cases (**Figure 1B**). To complement these findings, LSR transcript expression was analyzed using The Cancer Genome Atlas (TCGA) dataset. As depicted in **Figure 1C**, LSR mRNA levels were significantly elevated in TNBC compared to normal breast tissue. These transcriptomic data align with the TMA protein expression results, suggesting LSR as a promising therapeutic target. In addition to TNBC, the ER^+^/PR^+^ patients and HER2^+^ patients also showed high counts of LSR transcripts. Therefore, targeting LSR could cover and benefit multiple subtypes of breast cancer. LSR expression was further evaluated in TNBC cell lines, including human MDA-MB-231, human MDA-MB-468 and mouse 4T1. Western blot analysis revealed consistently high LSR expression across these three cell lines (**Figure 1D**), corroborating the TMA protein and TCGA transcript analyses. This study used MDA-MB-231 and 4T1 cell lines for *in vitro* anti-cancer cytotoxicity assays and *in vivo* anti-TNBC efficacy evaluations. Collectively, these results underscore LSR as a promising therapeutic target for TNBC and potentially other breast cancer subtypes.

### Development, engineering and production of anti-LSR mAb

**Figure 2:**
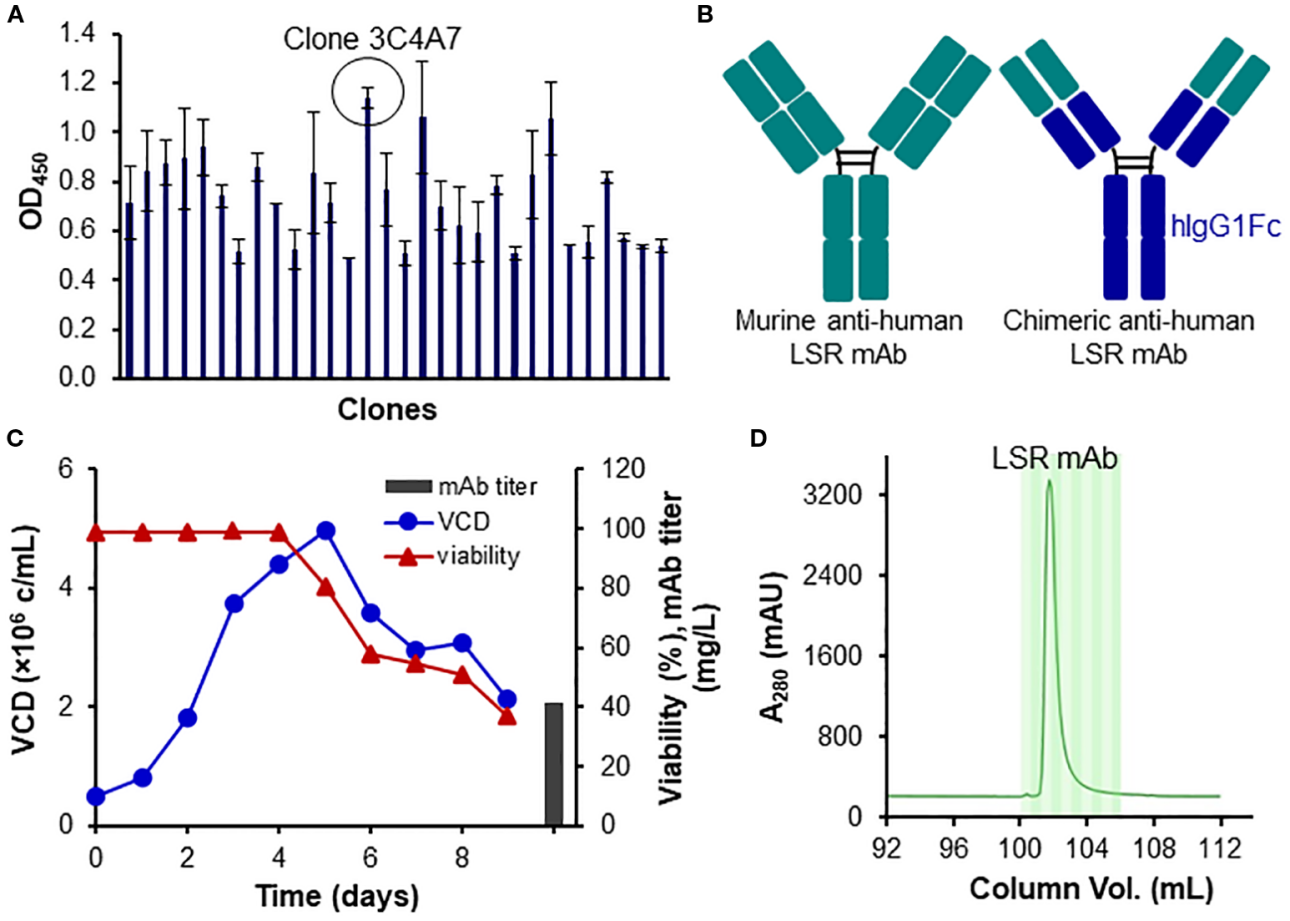
Development, engineering and production of LSR mAb. **(A)** Clone ranking of murine anti-human LSR mAb. **(B)** Engineering of LSR mAb by fusing the variable region of the murine anti-human LSR mAb with the constant region of human IgG. **(C)** Transient production of engineered LSR mAb using HEK293 cells. **(D)** Purification of engineered LSR mAb using protein A column with liquid chromatography.

We developed a new mAb to target the extracellular domain (Met1-Asp259) of human LSR (UniProtKB A0A8Z5ABK9). The top hybridoma clone 3C4A7, which had high titer and binding affinity for the extracellular domain of CTYQMTSTPTQPIV, was identified (**Figure 2A**). After sequencing, the top clone was further engineered by fusing its variable region with the Fc region of a human IgG1 (**Figure 2B**). The engineered LSR mAb was produced in fed-batch suspension culture using transient expression system. As shown in **Figure 2C**, 41.6 mg/L of mAb was produced from a 9-day culture with maximum viable cell density (VCD) of 4.9x10^6^ cells/mL. Glucose (4 g/L) and GlutaMAX (4 mM) were added to the production culture on Days 3 and 5. Purified mAb was generated from affinity purification using liquid chromatography system (**Figure 2D**).

### TNBC targeting and cross-species reactivity of LSR mAb

**Figure 3:**
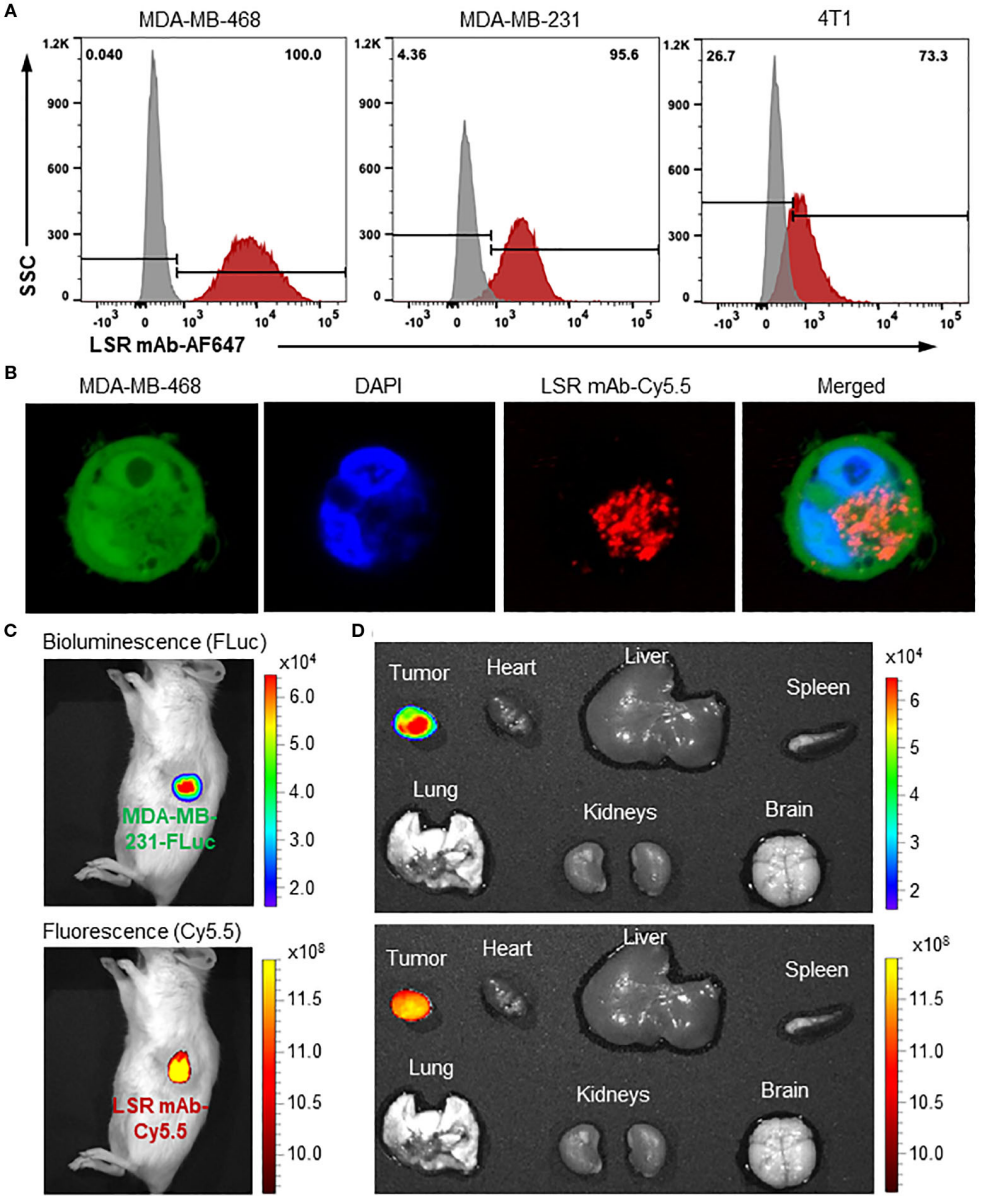
Evaluations of TNBC surface binding, internalization and specificity of anti-LSR mAb. **(A)** Flow cytometry analysis of LSR mAb surface binding to TNBC cells. **(B)** Confocal microscope showing internalization of mAb (Cy5.5, red) in MDA-MB-468 cells (GFP, green). **(C)** IVIS imaging of MDA-MB-231 xenografted NSG mice with administration of LSR mAb-Cy5.5. About 80 µg of mAb was injected via tail vein. **(D)** *Ex vivo* imaging with IVIS to validate TNBC specificity.

To evaluate the surface binding of our engineered anti-LSR mAb to TNBC cells, we performed flow cytometry on human TNBC cell lines (MDA-MB-231 and MDA-MB-468) and a murine TNBC cell line (4T1) (**Figure 3A**). The mAb exhibited high surface binding, with rates of 100% in MDA-MB-468, 95.6% in MDA-MB-231, and 73.3% in 4T1. The extracellular domain (residues 89-259) of human LSR (UniProt: Q86X29), used as the immunogen, shares 91% amino acid sequence similarity with the corresponding extracellular region (residues 1-171) of mouse LSR (UniProt: Q99KG5). This high homology accounts for the cross-species reactivity of our anti-human LSR mAb, though binding to murine TNBC cells was comparatively lower. The ability to target both human and murine LSR supports the use of mouse models for future preclinical studies. Additionally, IHC staining of TNBC patient tissues (**Figure 1A**) confirmed the specificity of our chimeric anti-LSR mAb for human TNBC.

Next, mAb internalization in TNBC cells was investigated using live-cell confocal microscopy. As presented in **Figure 3B**, the Cy5.5 labelled LSR mAb (displayed as red color) was detected in the cytoplasm of MDA-MB-468 cells expressing GFP (green color) 12 hours after incubation. As previously reported (33, 40, 47), the mAb is internalized via receptor-mediated endocytosis and trafficked to late endosomes for lysosomal degradation, which releases the conjugated cytotoxic drugs into the cytoplasm, causing cell death (48).

Finally, the MDA-MB-231-FLuc xenografted mouse models were established to assess the *in vivo* TNBC-specific targeting and biodistribution of the LSR mAb. Live-animal IVIS imaging displayed colocalization of the Cy5.5 fluorescence signal (mAb) with the FLuc bioluminescence signal (TNBC tumor) 24 hours after tail vein injection (**Figure 3C**). The *ex vivo* IVIS analysis confirmed strong accumulation of LSR mAb (Cy5.5) in the tumor, with no detectable signal in the brain, heart, lung, spleen, liver, and kidneys (**Figure 3D**). These results demonstrate that our engineered LSR mAb selectively targets TNBC *in vivo* with minimal off-target effects, highlighting its potential to effectively deliver therapeutic payloads for cancer treatment.

### *In vitro* cytotoxicity of LSR-targeting ADC

**Figure 4:**
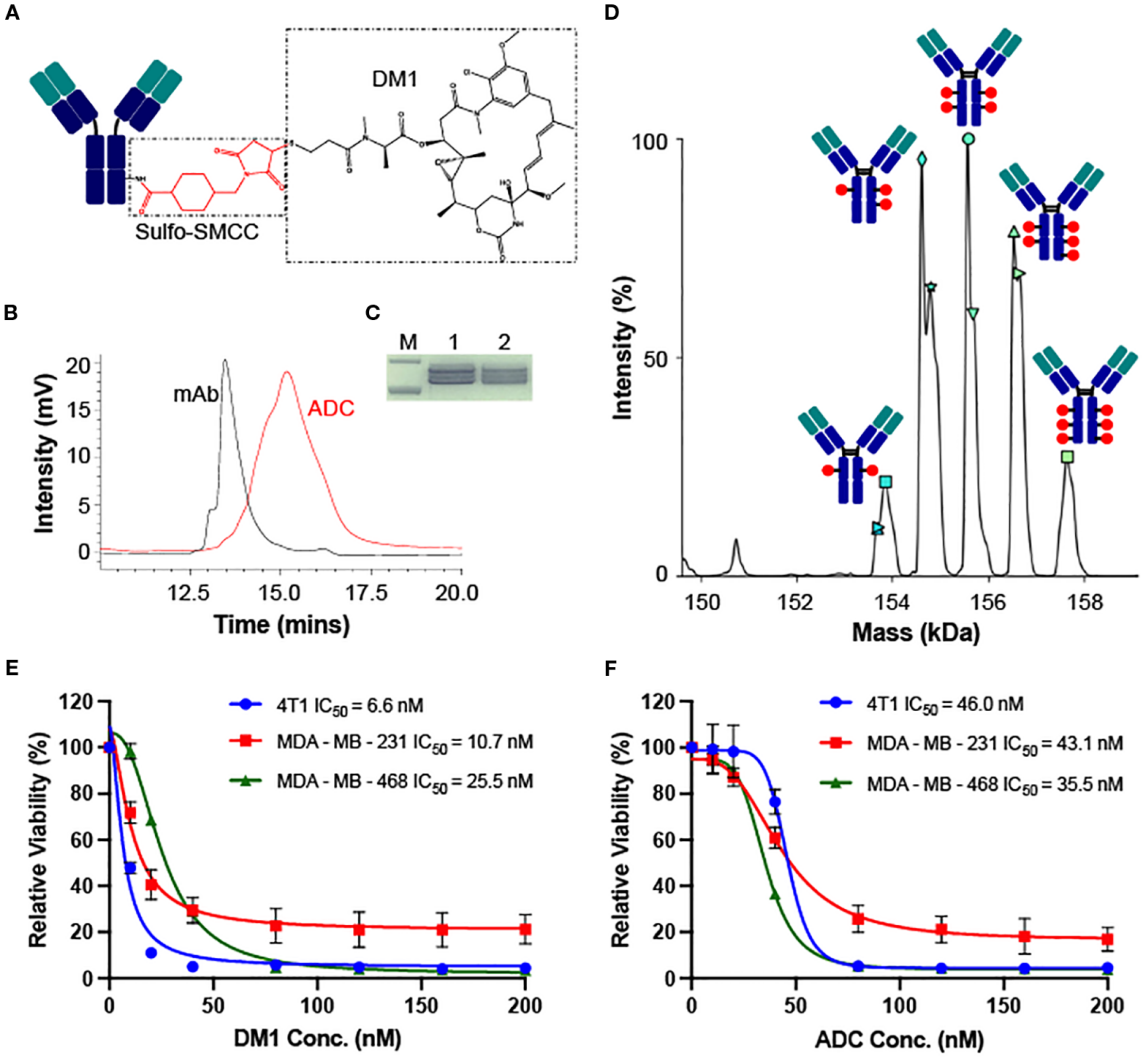
Construction, characterization and *in vitro* cytotoxicity of LSR ADC. **(A)** Structure of LSR mAb-DM1. **(B)** HPLC analysis to confirm conjugation of mAb and drug. **(C)** SDS-PAGE analysis of ADC integrity. M: Marker; 1: LSR mAb; 2: LSR mAb-DM1. **(D)** DAR (3.91) analysis of ADC using mass spectrometry. **(E)** Cytotoxicity of free DM1 in TNBC 4T1, MDA-MB-231 and MDA-MB-468. n = 3. **(F)** Cytotoxicity of LSR mAb-DM1 in three TNBC cell lines. n = 3.

The LSR-targeting ADC was constructed by conjugating the LSR mAb with DM1 via sulfo-SMCC linker (**Figure 4A**). HPLC characterization of ADC confirmed the homogenous conjugation of mAb and chemotherapy with a conversion rate of ~100% (**Figure 4B**). SDS-PAGE analysis verified the structural integrity of the LSR ADC (**Figure 4C**). The anti-TNBC cytotoxicity of free DM1 and LSR mAb-DM1 ADC was assessed in human and mouse TNBC cell lines. The mass spectrometry analysis showed that the drug to antibody ratio (DAR) of LSR ADC was 3.91 (**Figure 4D**). As presented in **Figure 4E**, free DM1 exhibited IC_50_ values of 6.6 nM (4T1), 10.7 nM (MDA-MB-231) and 25.5 nM (MDA-MB-468). The IC_50_ values for LSR mAb-DM1 ADC were 46.0 nM (4T1), 43.1 nM (MDA-MB-231) and 35.5 nM (MDA-MB-468) (**Figure 4F**). These cytotoxicity results illustrate that LSR-DM1 ADC can effectively kill TNBC cells *in vitro*. The ADC has lower cytotoxicity to TNBC cells than free drug at low concentrations of 10–40 nM, which can be explained by the multi-step (receptor binding, internalization, and drug release) targeted drug delivery process.

### Anti-TNBC efficacy in MDA-MB-231 xenograft models

**Figure 5:**
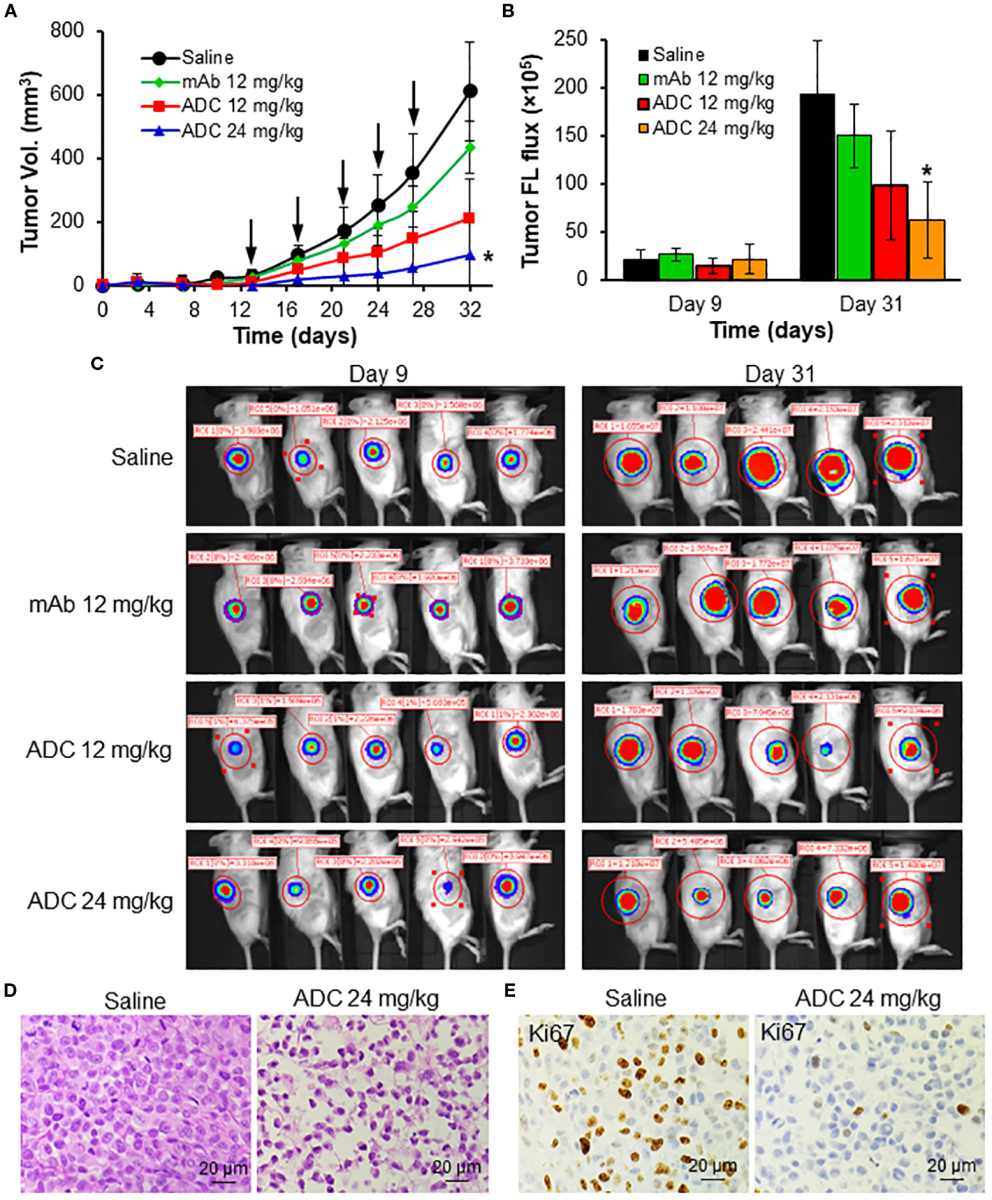
*In vivo* anti-TNBC efficacy of LSR ADC in MDA-MB-231-FLuc xenograft mouse models. **(A)** Tumor volume profile as indicated by black arrow. n = 6. Data were presented as mean ± SEM, *n* = 6. *P<0.05 *vs*. saline using ANOVA followed by Dunnett’s *t*-test. **(B)** Tumor fluorescence flux measured with IVIS. **(C)** IVIS imaging of mice on Days 9 and 31. **(D)** H&E staining of harvested tumors. Scale bar equals 20 µm. **(E)** IHC staining of tumor tissues with Ki67 antibody. Scale bar equals 20 µm.

The anti-tumor efficacy study was first conducted in human MDA-MB-231-FLuc xenografted NSG mice. As described in **Figure 5A**, the TNBC tumor volumes reached 612.2 mm^3^ (saline), 435.8 mm^3^ (12 mg/kg-BW LSR mAb), 212.4 mm^3^ (12 mg/kg LSR mAb-DM1 ADC), and 95.7 mm^3^ (24 mg/kg ADC) on Day 32. Compared to the saline group, LSR mAb reduced tumor burden by 29%, which could be attributed to the inhibition of lipid uptake or synthesis by the mAb. The ADC at 12 and 24 mg/kg reduced tumor burden by 65% and 85%, respectively, demonstrating dose-dependent tumor growth inhibition. TNBC tumor volume, assessed via FLuc flux at the end of treatment, corroborated that the 24 mg/kg ADC dose effectively suppressed TNBC growth (**Figure 5B**). The IVIS images, collected before treatment and on Day 31, also confirmed the ADC’s inhibitory effect on tumor growth (**Figure 5C**). H&E staining of paraffin-embedded tumor tissue sections revealed reduction of tumor intensity in ADC treatment groups, with no evident cancer cell death in the saline group (**Figure 5D**). IHC staining with anti-Ki67 antibody confirmed that ADC treatment significantly inhibited TNBC cell proliferation (**Figure 5E**).

### Anti-TNBC efficacy in 4T1 xenograft models

**Figure 6:**
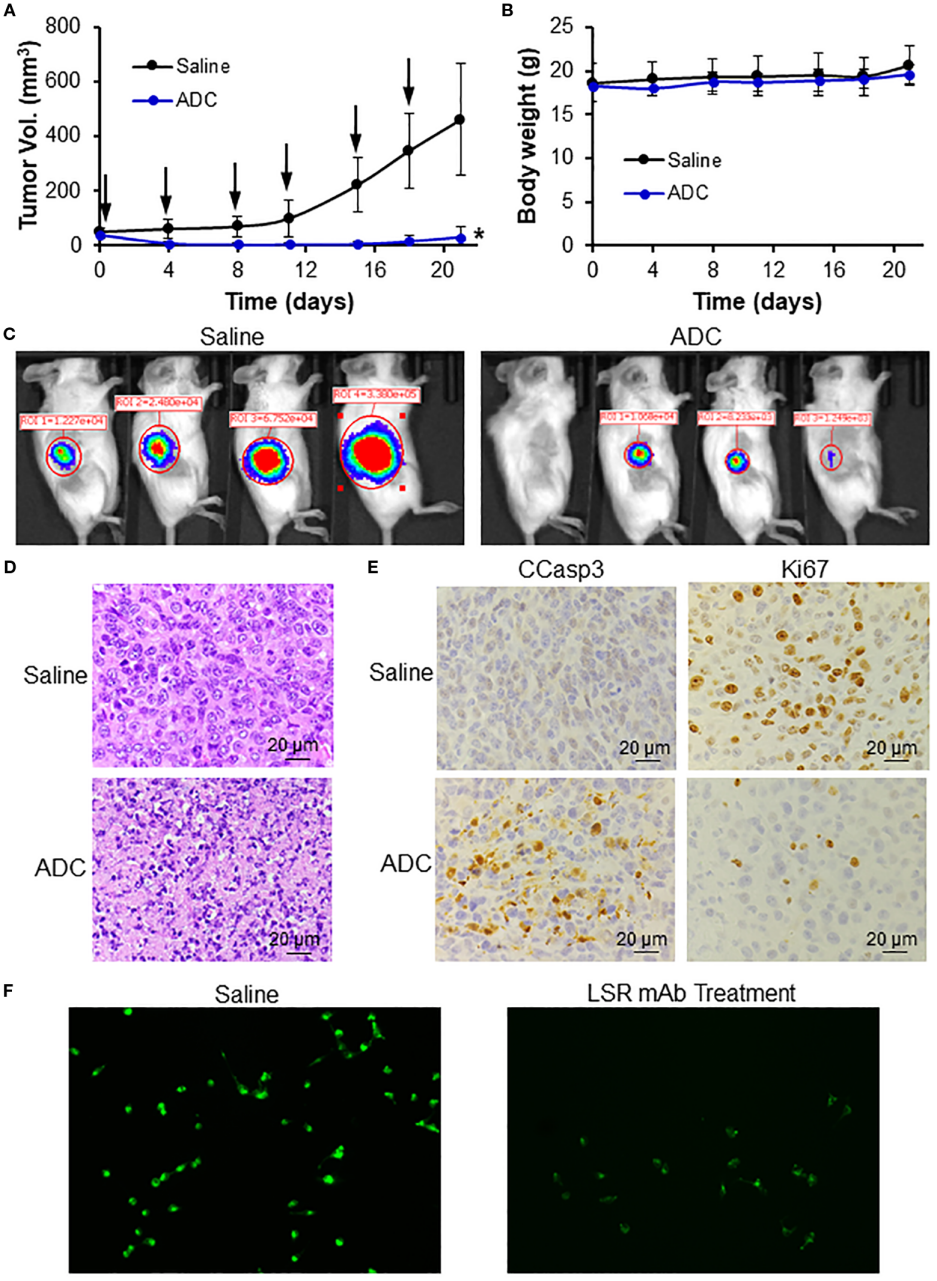
*In vivo* anti-TNBC efficacy of LSR ADC in 4T1-Fluc xenograft mouse models. **(A)** Tumor volume profiles. Black arrow indicating i.v. injection. n = 4. Data were presented as mean ± SEM, *n* = 6. *P<0.05 *vs*. saline using ANOVA followed by Dunnett’s *t*-test. **(B)** Body weight. **(C)** IVIS imaging of mice on Day 17. **(D)** H&E staining of tumor tissue indicating apoptosis and cell death of TNBC in treatment group. Scale bar equals 20 µm. **(E)** IHC staining of tumor tissues with Ki67 (cell proliferation inhibition) and CCasp3 (apoptosis) antibodies. Scale bar equals 20 µm. **(F)** LSR mAb reduce lipid droplets (green) in MDA-MB-231 cells.

The anti-TNBC efficacy of the LSR mAb-DM1 ADC was validated in immunocompetent BALB/cJ mice bearing 4T1 xenografts. Treatment with 24 mg/kg ADC significantly reduced tumor volume and caused tumor shrinkage (**Figure 6A**), without affecting mouse body weight, suggesting minimal systemic toxicity (**Figure 6B**). Live-animal IVIS imaging confirmed the effectiveness of ADC treatment (**Figure 6C**), as indicated by the undetectable or weak FLuc signal in the treatment group. H&E staining of tumor tissues harvested at the end of the study revealed severe tumor cell death in the treatment group (**Figure 6D**). The tumor volume slightly increased on Days 18–20 in **Figure 6A**, but the dead cells in H&E staining indicated that this increase could be caused by non-cellular factors (e.g., inflammation, vascular formation, necrosis) instead of tumor growth recurrence. IHC staining of tumor sections showed that the ADC induced apoptosis, as indicated by the upregulation of cleaved caspase 3, and inhibited cancer cell proliferation, as displayed by the downregulation of Ki67 (**Figure 6E**). Live-cell confocal microscopy demonstrated that anti-LSR mAb reduced lipid uptake (green fluorescence) in MDA-MB-231 cells (**Figure 6F**), consistent with literature reports that LSR promotes lipid uptake and fatty acid oxidation in gastric cancer (29, 49).

### Evaluation of toxicity

**Figure 7:**
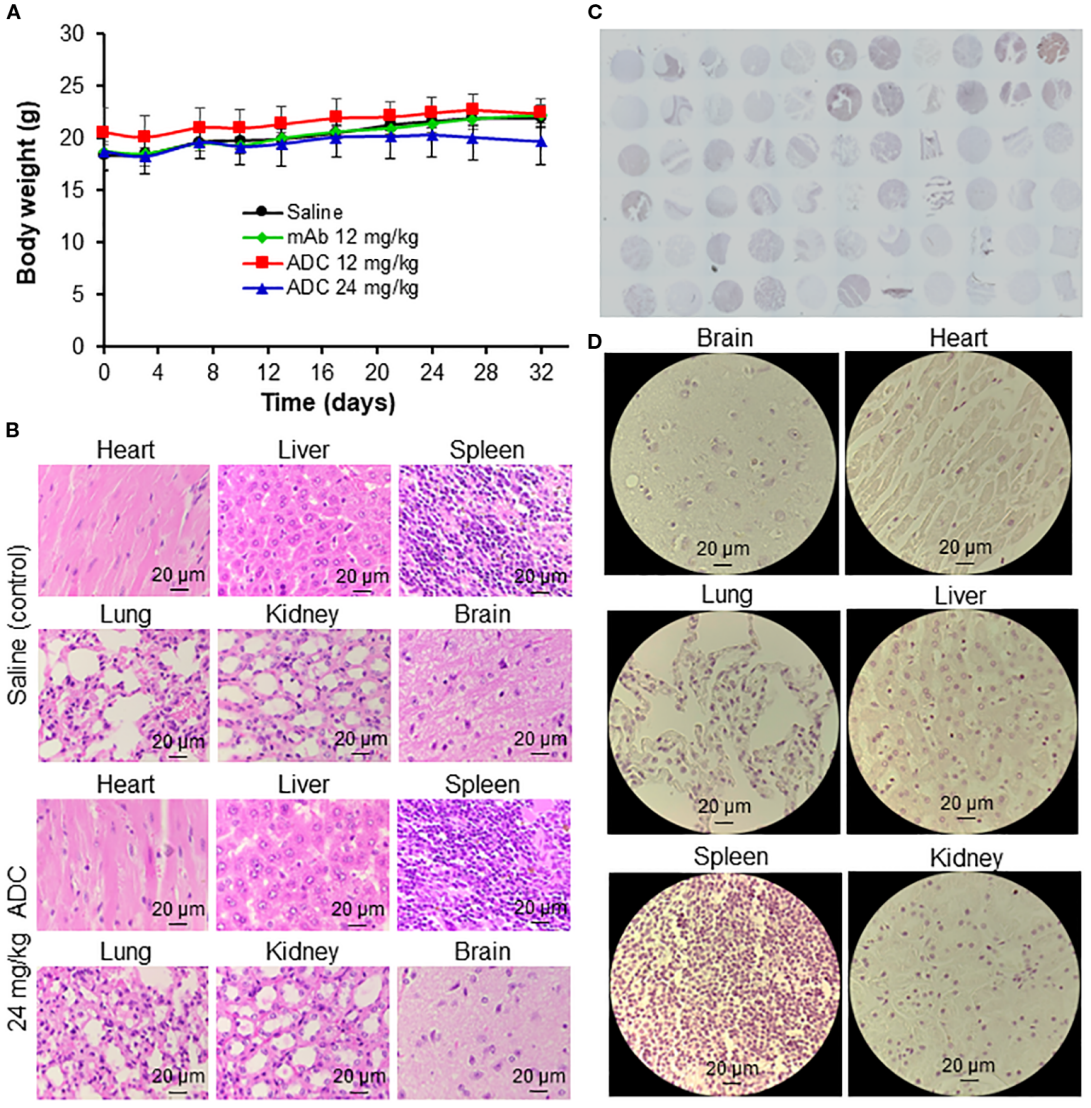
Evaluation of off-target and toxicity of LSR ADC. **(A)** Body weight of mice. n = 6. **(B)** H&E staining of major organs (heart, liver, spleen, lung, kidney, and brain). Scale bar equals 20 µm. **(C)** IHC staining of 33 human normal organs, including cerebrum, cerebellum, peripheral nerve, adrenal gland, thyroid gland, spleen, thymus, bone marrow, lymph node, tonsil, pancreas, liver, esophagus, stomach, small intestine, colon, lung, salivary, pharynx, kidney, bladder, testis, prostate, penis, ovary, uterine tube, breast, endometrium, cervix, cardiac muscle, skeletal muscle, mesothelium and skin. n = 2. **(D)** Representative IHC images of major organs. Scale bar equals 20 µm.

In animal studies, ADC treatment at 12 and 24 mg/kg did not cause body weight loss (**Figure 7A**), indicating low systemic toxicity. H&E staining of major organs (heart, brain, lung, liver, kidney, spleen) showed no signs of inflammation, apoptosis, necrosis, or damage in the mAb or ADC groups (**Figure 7B**). IHC staining of 33 normal human tissues (n = 2) with the engineered LSR mAb revealed no significant off-target binding, as confirmed by whole-slide scanning (**Figure 7C**). High-resolution images of heart, brain, lung, liver, kidney, and spleen showed no significant mAb binding (**Figure 7D**). These findings suggest that the LSR mAb selectively targets LSR-overexpressing TNBC cells with minimal off-target effects on normal tissues.

## Discussion

The absence of effective cell surface receptors in TNBCs has hindered the development of new targeted therapies. Recently, Trop-2 and CD276 (47, 50–53) have emerged as promising therapeutic targets for TNBC and other cancers. Trop-2-targeting sacituzumab govitecan (54–56) and datopotamab deruxtecan (57–59) have been employed to treat breast and other malignancies, but their clinical efficacy remains limited in recurrent and metastatic settings. Recent clinical trials of GSK5764227 (HS-20093) and Ifinatamab deruxtecan (NCT06203210, NCT04145622) (50, 60) have evaluated their potential for treating prostate cancer and small cell lung cancer treatment, but trials in TNBC patients has not yet begun. Importantly, the extreme heterogeneity of TNBCs necessitates the exploration of additional surface markers to ensure broader therapeutic coverage. This study identifies LSR as an alternative surface receptor. Our future study will evaluate dual targeting strategies, such as incorporating LSR together with Trop-2 or CD276. The efficacy of LSR-targeted therapy was validated in TNBC mouse models, confirming its therapeutic potential. Beyond TNBC, LSR exhibits high transcript expressions in ER^+^/PR^+^ and HER2^+^ breast cancer subtypes, suggesting its therapeutic potential across multiple breast cancer types.

The anti-LSR mAb developed in this study shows significant promise as a targeted delivery vehicle for potent payloads. The mAb demonstrated high surface binding to TNBC cells *in vitro* and selective tumor targeting *in vivo*, with minimal off-target effects in normal human and mouse organs, making it an ideal candidate to target TNBC. The engineering and construction of the chimeric LSR mAb further enhance its future clinical value, with the expectation of improved circulation stability. Additionally, the anti-LSR ADC conjugated with a standard cytotoxic microtubule inhibitor DM1 significantly reduced tumor burden in two TNBC xenografted mouse models. No body weight loss or detectable systematic toxicity was observed, suggesting manageable side effects in future clinical applications. Notably, the mAb exhibited minimal binding to 33 normal human organs, with only weak signals in human liver and pancreas tissues, despite reported high physiological LSR expression in hepatocytes (61, 62). This selectivity may stem from differences in post-translational modifications (PTMs) between tumors and normal cells, which could affect mAb binding efficacy. Further analysis of PTMs and their impact on antibody binding are needed in future study to elucidate the mechanism underlying this cancer-specific targeting. Moreover, the anti-LSR mAb demonstrated modest therapeutic potential as a standalone agent, slightly reducing tumor growth and inhibiting lipid uptake and synthesis.

The LSR-targeted ADC significantly inhibited TNBC tumor growth in two xenograft mouse models. However, while tumor growth was completely suppressed in syngeneic models, 15% residual tumor volume persisted in immunocompromised models. Several factors may contribute to this partial efficacy, including suboptimal dosing, reliance on a single payload (DM1), or relapse of surface receptor expression in some tumor cells. Recent studies suggest LSR suppresses CD8^+^ T-cell activity in the tumor microenvironment (63), which may explain the enhanced efficacy in syngeneic models, where the ADC’s cytotoxic effects (via DM1) synergize with LSR mAb-mediated immune activation. Nevertheless, comprehensive mechanistic studies are essential in future research to validate this hypothesis. For example, advanced immunocompetent models, such as patient-derived xenografts, could elucidate these synergistic anti-cancer mechanisms.

To overcome these challenges and enhance TNBC treatment effectiveness, the following strategies could be implemented. First, the optimal dose and treatment schedule of LSR ADC can be identified from pharmacokinetics studies. Second, a dual-payload ADC platform, conjugating synergistic anti-cancer agents (e.g., cytotoxic and immune-enhancing payloads), could improve the anti-cancer efficacy. Dual targeting of receptors like CD276 and LSR may expand patient coverage and mitigate receptor relapse-driven resistance. Additionally, advanced cleavable linkers with high plasma stability and cancer-specific payload release could enhance drug delivery efficiency. Finally, combining LSR ADC with synergistic therapies, such as immunotherapy or gene therapy, could improve tumor clearance and reduce resistance risk.

High LSR expression correlates with poor prognosis in gastric (49, 64), epithelial ovarian (65), and endometrial cancers (66), suggesting the anti-LSR mAb and ADC may have broader applications across multiple cancer types. While LSR’s role in cancer remains incompletely understood, our anti-LSR mAb offers a valuable tool to investigate its contributions to tumorigenesis, metabolic reprogramming (67), and immune regulation (63, 68) in future studies. Combining LSR-targeted ADC with existing therapeutic regimens could yield synergistic effects.

Moving forward, we aim to develop dual-payload ADCs using our engineered LSR mAb, combining chemotherapy and immunotherapy for enhanced anti-TNBC efficacy, stability, and functionality. Dual-receptor targeting will be explored to broaden treatment coverage. The anti-LSR ADC will also be evaluated in clinically relevant models, such as patient-derived xenografts in humanized mice, non-human primates, or organ-on-chip systems, to generate preclinical data for future clinical trials.

## Data Availability

The raw data supporting the conclusions of this article will be made available by the authors, without undue reservation.
